# Risk of pancreatitis after pancreatic duct guidewire placement during endoscopic retrograde cholangiopancreatography

**DOI:** 10.1371/journal.pone.0190379

**Published:** 2018-01-10

**Authors:** Yuki Ishikawa-Kakiya, Masatsugu Shiba, Hirotsugu Maruyama, Kunihiro Kato, Shusei Fukunaga, Satoshi Sugimori, Koji Otani, Shuhei Hosomi, Fumio Tanaka, Yasuaki Nagami, Koichi Taira, Hirokazu Yamagami, Tetsuya Tanigawa, Toshio Watanabe, Yasuhiro Fujiwara

**Affiliations:** Department of Gastroenterology, Osaka City University Graduate School of Medicine, Osaka, Japan; University of Szeged, HUNGARY

## Abstract

**Background & aims:**

Advanced techniques have been developed to overcome difficult cannulation cases in endoscopic retrograde cholangiopancreatography (ERCP). Pancreatic duct guidewire placement method (PGW) is performed in difficult cannulation cases; it is possible that it places patients at risk of post-ERCP pancreatitis (PEP). The mechanism of PEP is still unclear, but pancreatic duct pressure and injury of pancreatic duct are known causes of PEP. Therefore, we hypothesized a relationship between pancreatic duct diameter and PEP and predicted that PGW would increase the risk of PEP in patients with non-dilated pancreatic ducts. This study aimed to investigate whether PGW increased the risk of PEP in patients with pancreatic duct diameter ≤ 3 mm.

**Methods:**

We analyzed 332 patients with pancreatic duct ≤ 3 mm who performed first time ERCP session. The primary endpoint was the rate of adverse event of PEP. We evaluated the risk of PEP in patients who had undergone PGW compared to those who had not, using the inverse probability of treatment weighting (IPTW) analysis.

**Results:**

PGW was found to be an independent risk factor for PEP by univariate analysis (odds ratio [OR], 2.45; 95% confidence interval [CI], 1.12–5.38; p = 0.03) after IPTW in patients with pancreatic duct diameter ≤ 3 mm. Adjusted for all covariates, PGW remained an independent risk factor for PEP (OR, 3.12; 95% CI, 1.33–7.33; p = 0.01).

**Conclusion:**

Our results indicate that PGW in patients with pancreatic duct diameter ≤ 3 mm increases the risk of PEP.

## Introduction

Endoscopic retrograde cholangiopancreatography (ERCP) is an important procedure for the diagnosis and treatment of pancreatic biliary disease, but post-ERCP pancreatitis (PEP) is the most common adverse event, which can lead to death [1–3]. PEP is associated with cannulation difficulties, as prolonged and repeated cannulation attempts may injure the papilla [4, 5]. Therefore, many selective bile duct cannulation techniques have been developed to prevent PEP [6]. The pancreatic guidewire placement method (PGW) is one such technique, reported by Dumonceau in a patient who underwent Billroth I anastomosis [7]. The European Society of Gastrointestinal Endoscopy’s clinical guidelines recommend using PGW in difficult cannulation patients[8]; however, PGW may increase the risk of PEP [9]. According to randomized trials, there is no risk of PEP with PGW compared to conventional cannulation techniques [10, 11]; however, Sasahira *et al*. reported that leaving the guidewire in the pancreatic duct might cause irritation and injury of the pancreatic duct and parenchyma [12]. Therefore, the association between PGW and PEP is controversial, and it is necessary to clarify the effect of PGW on the risk of PEP.

The mechanism of PEP is still unclear and is considered to be related to various factors, including pancreatic duct pressure and damage to the pancreatic duct. However, no existing studies have focused on the relationship between pancreatic duct diameter and PEP. We focused on pancreatic duct diameter and PGW to clarify the association between PGW and PEP, and hypothesized that when the pancreatic duct diameter is not dilated, PGW may increase the risk of PEP by further increasing the pressure in the pancreatic duct, or by causing irritation or injury to the duct during ERCP. Therefore, we focused on patients with pancreatic duct diameter of ≤ 3 mm. This is the first study to determine the risk of PEP by focusing on pancreatic duct diameter. The inverse probability of treatment weighting (IPTW) technique was used based on the propensity score to reduce selection bias.

The aim of this study was to clarify whether PGW increases the risk of PEP in patients with a pancreatic duct diameter of ≤ 3 mm, by conducting a retrospective, comparative study using the IPTW technique.

## Methods

### Study design and patients

#### Patient recruitment and data collection

A retrospective review of all patients with naive papilla who underwent first-time ERCP for biliary intervention at our department in Osaka City University hospital between January 2010 and December 2015 was conducted. All patients provided written informed consent for the use of personal data. The protocol of this study was approved by the Ethics Committee of the Osaka City University Graduate School of Medicine (No.3659) and registered in UMIN (UMIN000026416). Inclusion criteria included patients with a pancreatic duct diameter of ≤ 3 mm. This study’s purpose was to evaluate whether PGW increased the risk of PEP compared to other bile duct insertion methods in patients with a pancreatic duct diameter ≤ 3 mm. Therefore, the exclusion criteria was determined as failed cannulation of the bile duct or pre-cannulation perforation. Further, pancreatic duct stenting could significantly lower the risk of PEP; thus, the exclusion criteria comprised pancreatic duct stenting.

Using patients’ electronic medical records, age, sex, body mass index (BMI), and pancreatic duct diameter, and whether patients had undergone endoscopic retrograde pancreatography (ERP), precut sphincterotomy, intraductal ultrasonography (IDUS), endoscopic sphincterotomy (EST), endoscopic papillary balloon dilatation (EPBD), endoscopic papillary large-balloon dilation (EPLBD), bile duct stone removal, or bile duct brush cytology, bile duct biopsy, or experienced adverse events were recorded. The endoscopist and type of endoscope used was also recorded.

#### Measure of pancreatic duct diameter

The maximum diameter of the pancreatic duct was measured by using examination images: non-contrasted computed tomography (CT), enhanced CT, and magnetic resonance imaging (MRI). All images were taken within three months before ERCP. A pancreatic duct diameter greater than 3 mm was defined as dilated, as detailed in several reports [13–15].

#### Endoscopic procedure

All patients undergoing ERCP were administered an intravenous injection of midazolam (3–10 mg) and pentazocine (15 mg), the dose was depending on age and tolerance. The procedures were carried out with a side-viewing duodenoscope (JF240, JF260V, TJF240, TJF260V; Olympus Optical Corporation, Tokyo, Japan) by expert endoscopists or trainees who had been performing ERCP for fewer than 5 years. Although the use of prophylactic antibiotics and gabexate mesilate were controversial, they were still administered routinely to all patients to prevent cholangitis and pancreatitis. No patients used prophylaxis of NSAIDs suppository before or after ERCP, because prophylaxis of NSAIDs in Japan was not reimbursed by insurance company currently. Standard cannulation or wire-guided cannulation of the bile duct, using a 0.035-inch or 0.025-inch guidewire (Hydra Jagwire; Boston Scientific, Ireland or VisiGlide 2; Olympus, Tokyo, Japan), was initially performed. After the guidewire was inserted into the ampulla, routine cholangiography was performed using a cannula (MTW Endoskopie; Wesel, Germany) or sphincterotome (CleverCut3; Olympus, Tokyo, Japan), as decided by the endoscopist. If the bile duct was visualized, the procedure was continued. All patients were hospitalized for at least 72 hours after the procedure. Serum amylase levels were measured at 4 and 24 hours after ERCP. Abdominal CT was performed if needed.

#### Pancreatic guidewire placement method

The indication for PGW-placement was determined during the procedure according to the clinical judgment of the endoscopists. A 0.035-inch or 0.025-inch guidewire was inserted (Hydra Jagwire; Boston Scientific, Ireland or VisiGlide 2; Olympus, Tokyo, Japan) into the pancreatic duct, and its position was monitored using fluoroscopy or an injection contrast agent. Subsequently, second cannula pre-loaded with guidewire was passed into the same working channel of the scope beside the first pancreatic guidewire. The tip of the cannula then carefully manipulated through the papilla over the pancreatic guidewire and was inserted into the bile duct.

#### Primary endpoint and evaluation of post-ERCP pancreatitis

Primary endpoint was the rate of adverse event of PEP in patients with pancreatic duct diameter of ≤ 3 mm.

Following the definition given by Cotton’s report[1], we defined PEP as a medical condition with abdominal pain persisting for at least 24 hours after the procedure and high serum amylase (> 360 IU/L) greater than threefold the upper normal limit at 4 to 24 hours after the procedure. Elevation of serum amylase with no development of abdominal pain was defined as post-ERCP hyperamylasemia. Regarding the severity of PEP, we modified Cotton’s definition with reference to other papers[16] because post-ERCP patients usually stayed in the hospital about 4–5 days. Therefore, the severity of PEP was classified according to the period of fasting required for PEP. Mild PEP required 2–3 days; moderate PEP required 4–10 days; and severe PEP required more than 10 days, necessitated surgical or intensive treatment, or contributed to death. The primary outcome of this study was PEP, including mild, moderate, and severe PEP.

### Statistical analysis

Continuous variables are presented as mean ± standard deviation, or as median ± interquartile range; categorical variables are presented as numbers. The data were evaluated using unpaired t-tests (continuous variables) or the chi-squared test (categorical variables); if the sample size was too small, Fisher’s exact test was used to analyze categorical variables. Univariate and multivariate logistic regression analyses were performed to identify factors associated with PEP, then factors known risk factors of PEP (sex, ERP, precut sphincterotomy) were analyzed in the multivariate analysis. The model included age, sex, BMI, pancreatic duct diameter, ERP, precut sphincterotomy, IDUS, EST, EPBD, EPLBD, bile duct stone removal, bile duct brush cytology, bile duct biopsy, PGW, endoscopist, and type of endoscope. For each factor considered to have a potential association with PEP, the odds ratio (OR) with its 95% confidence interval (95% CI) was calculated.

Further, IPTW based on propensity scores was used to reduce selection bias by creating a ‘pseudo-population’ in this study. IPTW was calculated by the inverse of the conditional probability of receiving the exposure that patients actually received [17, 18]. The above variables were used to generate a propensity score by logistic regression analysis. The validity of the model was assessed using c-statistics by estimating the area under the receiver operating characteristics curve. The reliability of the model was evaluated using the Hosmer-Lemeshow goodness-of-fit statistical analysis.

Statistical analyses were performed using SPSS^™^ software version 21.0 for Windows (SPSS Inc., Japan) and the R^™^ statistical package V.2.13.0 (http://www.r-project.org). All statistical tests were two-sided, and *p*-values < 0.05 were considered significant.

## Results

### Baseline characteristics of patients

**Fig 1:**
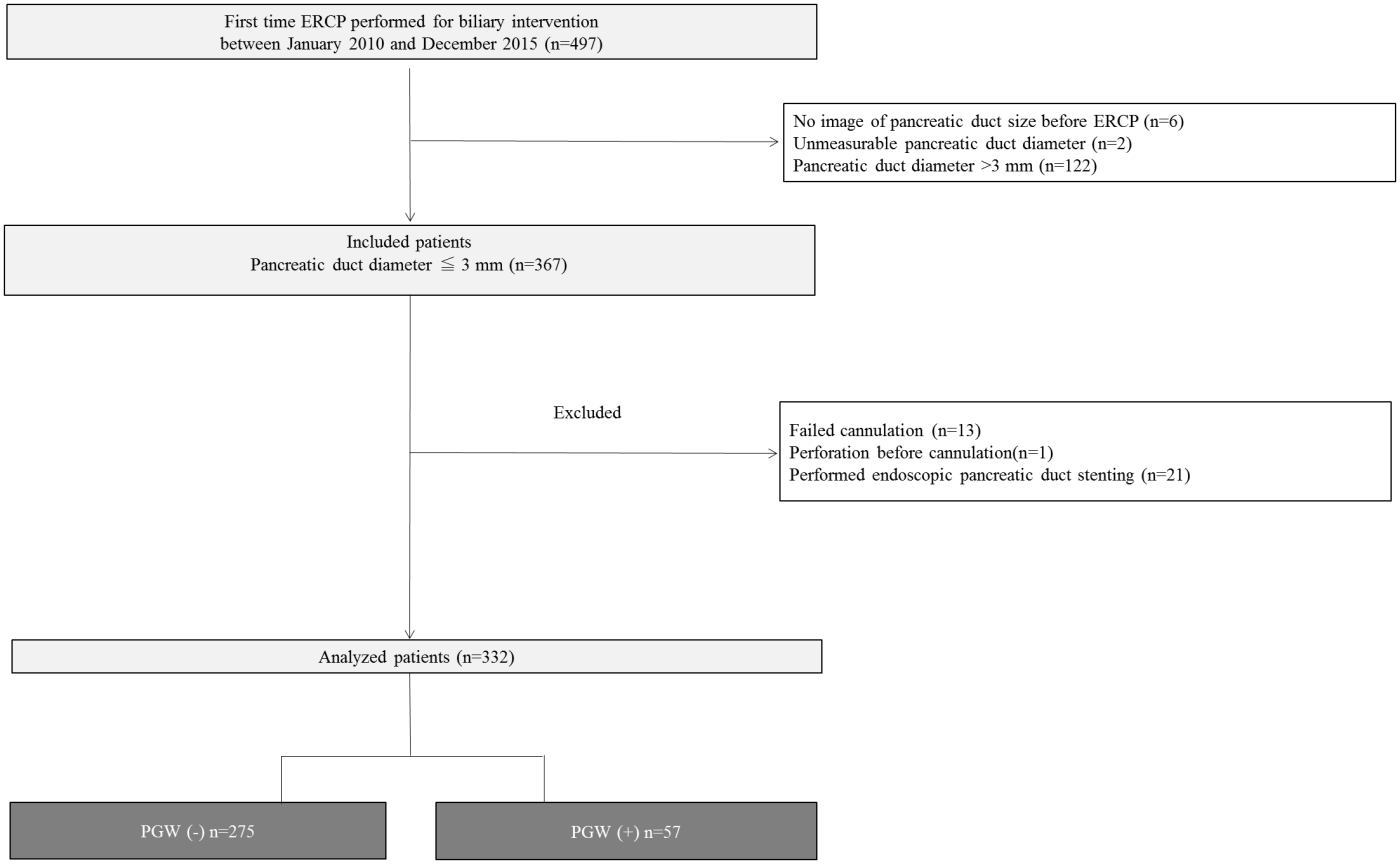
Diagram of the study design. ERCP, endoscopic retrograde cholangiopancreatography PGW, pancreatic guidewire placement method.

We collected the data of 497 patients with a naive papilla who had undergone first-time ERCP for biliary intervention, and included 367 patients who had a pancreatic duct diameter ≤ 3 mm. Thirty-five patients were then excluded because of endoscopic pancreatic duct stenting (n = 21), failed cannulation (n = 13), and perforation before cannulation (n = 1). We enrolled a total of 332 patients (men/women = 195/137; mean age = 67.9 years) in this study (Fig 1 and S1 Data). Table 1 shows the clinical characteristics of the patients. Patients were classified into two groups; 57 patients in the PGW (+) group, and 275 patients in the PGW (-) group. There was no significant difference between the PGW (-) group and (+) group regarding clinical variables.

**Table 1:** Baseline characteristics before IPTW.

		Before IPTW (n = 332)		
		PGW (-) n = 275, (%)	PGW (+) n = 57, (%)	*p* value
Age		67.59 ± 13.137	69.61 ± 11.129	0.28
Sex	male	156 (56.7)	39 (68.4)	0.11
BMI		22.24 ± 3.86	22.94 ± 3.83	0.22
Pancreatic duct diameter		1.83 ± 0.53	1.80 ± 0.54	0.76
ERP	yes	31 (11.3)	7 (12.3)	0.82
Precut sphincterotomy	yes	10 (3.6)	4 (7.0)	0.27
IDUS	yes	89 (32.4)	13 (22.8)	0.21
EST	yes	106 (38.5)	25 (43.9)	0.46
EPBD	yes	6 (2.2)	0 (0)	0.22
EPLBD	yes	11 (4.0)	0 (0)	0.45
Bile duct stone removal	yes	91 (33.1)	22 (38.6)	0.6
Bile duct brushing	yes	65 (23.6)	14 (24.6)	0.87
Bile duct biopsy	yes	25 (9.1)	4 (7.0)	0.8
Scopist	expert	66 (24.0)	13 (22.8)	1
Scope	TJF	167 (60.7)	36 (63.2)	0.77
Pancreatitis		31 (11.3)	12 (21.1)	0.05
mild		17 (6.2)	8 (14.0)	0.05
moderate		10 (3.6)	4 (7.0)	0.43
severe		4 (1.5)	0 (0)	1
Hyperamylasemia		33 (12)	7 (12.3)	1
Perforation		2 (0.7)	0 (0)	1

Table 1 shows the clinical characteristics of the patients.

BMI, body mass index; ERP, endoscopic retrograde pancreatography; IDUS, intraductal ultrasonography; EST, endoscopic sphincterotomy; EPBD, endoscopic papillary balloon dilatation; EPLBD, endoscopic papillary large-balloon dilation; IPTW, inverse probability of treatment weighting; PGW, pancreatic guidewire placement method

### Adverse events among the study subjects

The incidence rate of adverse events was 13.6%. In the PGW (-) group, PEP occurred in 31 patients (11.3%): mild PEP in 17 patients, moderate PEP in 10 patients, and severe PEP in 4 patients. In the PGW (+) group, PEP occurred in 12 patients (21.1%): mild PEP in 8 patients, moderate in 4 patients, and severe in 0 patients.

There were two adverse events of perforation in the PGW (-) group and no perforation in the PGW (+) group. Both perforations occurred after ERCP and both patients were treated surgically. The perforations were confirmed by surgery on the duodenum bulb and common bile duct.

### The risk of PEP

Table 2 shows the risk of PEP in the PGW (+) and PGW (-) groups. Univariate analysis showed that PGW increased the risk of PEP (OR, 2.10; 95% CI, 1.00–4.39; p = 0.049). Multivariate analysis also showed that the risk of PEP was significantly higher in the PGW (+) group than the PGW (-) group (OR, 2.22; 95% CI, 1.10–4.70; p = 0.04).

**Table 2:** Univariate and multivariate conditional logistic regression analysis of PEP.

			Before IPTW (n = 332)		Before IPTW (n = 332)	
	PEP (-) n = 289	PEP (+) n = 43	Crude OR (95% CI)	*p* value	Multivariate OR (95% CI)	*p* value
Age			0.10 (0.03–1.02)	0.8		
Sex (male)	172	23	0.78 (0.41–1.49)	0.46	0.73 (0.38–1.41)	0.36
BMI			0.99 (0.91–1.09)	0.87		
Pancreatic duct diameter			0.98 (0.56–1.71)	0.94		
ERP	31	7	1.62 (0.66–3.95)	0.29	1.60 (0.65–3.94)	0.31
Precut sphincterotomy	13	1	0.51 (0.06–3.97)	0.52	0.45 (0.06–3.56)	0.45
IDUS	89	13	0.97 (0.49–1.96)	0.94		
EST	117	14	0.71 (0.36–1.41)	0.32		
EPBD	4	2	3.48 (0.62–19.58)	0.16		
EPLBD	8	3	2.63 (0.67–10.34)	0.17		
Bile duct stone removal	98	15	1.04 (0.53–2.05)	0.9		
Bile duct brushing	67	12	1.28 (0.62–2.64)	0.5		
Bile duct biopsy	25	4	1.08 (0.36–3.28)	0.89		
PGW	45	12	2.10 (1.00–4.39)	0.049	2.22 (1.10–4.70)	0.04
Expert scopist	72	7	0.59 (0.25–1.37)	0.22		
Type of scope	176	27	1.08 (0.56–2.10)	0.81		

Table 2 shows the risk of PEP in the PGW (+) and PGW (-) groups.

BMI, body mass index; ERP, endoscopic retrograde pancreatography; IDUS, intraductal ultrasonography; EST, endoscopic sphincterotomy; EPBD, endoscopic papillary balloon dilatation; EPLBD, endoscopic papillary large-balloon dilation; IPTW, inverse probability of treatment weighting; PGW, pancreatic guidewire placement method; OR, Odds Ratio; CI, confidence interval

### Evaluation of inverse probability of treatment weighting

We created a quasi-randomized experiment using IPTW; that is, the subjects were randomly assigned to each group and were therefore equally likely to be in the PGW (-) group as in the PGW (+) group. The propensity-weighted model was well calibrated (Hosmer-Lemeshow test, p = 0.58).

Univariate and multivariate analyses for risk factors of PEP are shown in Table 3. In Table 3a, it is shown that in patients with a pancreatic duct diameter ≤ 3 mm, PGW was an independent risk factor for PEP by univariate analysis (OR, 2.45; 95% CI, 1.12–5.38; p = 0.03) after IPTW. Adjusted for sex, ERP, and precut, and all factors associated with PEP, PGW remained a risk factor for PEP (OR, 2.58; 95% CI, 1.16–5.78; p = 0.02); adjusted for all covariates, PGW was also an independent risk factor for PEP by multivariate analyses (OR, 3.12; 95% CI, 1.33–7.33; p = 0.01). We also analyzed another population that included 108 individual patients who had a pancreatic duct diameter > 3 mm and had not received a pancreatic duct stent as “another group” (Table 3b and S2 Data). There was no significant difference between the PGW (-) group and the PGW (+) group regarding PEP.

**Table 3:** The clinical factors for PEP before and after propensity score weighted by multivariate conditional logistic regression analysis. 3a: The model of patients with ≤ 3mm pancreatic diameter. 3b: The model of patients with > 3mm pancreatic diameter.

Table 3a				
	Before IPTW		After IPTW	
	Odds Ratio (95% CI)	*p* value	Odds Ratio (95% CI)	*p* value
Unadjusted	2.10 (1.00–4.39)	0.049	2.45 (1.12–5.38)	0.03
Adjusted for sex, ERP, precut sphincterotomy	2.22 (1.05–4.70)	0.04	2.58 (1.16–5.78)	0.02
Adjusted all covariates	2.53 (1.13–5.66)	0.02	3.12 (1.33–7.33)	0.01
Table 3b				
	Before IPTW		After IPTW	
	Odds Ratio (95% CI)	*p* value	Odds Ratio (95% CI)	*p* value
Unadjusted	2.13 (0.51–8.91)	0.3	0.94 (0.20–4.42)	0.94
Adjusted for sex, ERP, precut sphincterotomy	2.21 (0.52–9.37)	0.28	0.98 (0.20–4.77)	0.98
Adjusted all covariates	1.47 (0.19–11.46)	0.71	1.19 (0.11–12.61)	0.89

Table 3 shows univariate and multivariate analyses for risk factors of PEP.

IPTW, inverse probability of treatment weighting; ERP, endoscopic retrograde pancreatographycapsule endoscopy; CI, confidence interval

## Discussion

In the present study, PGW was clarified as an independent risk factor for PEP in patients with a pancreatic duct diameter of ≤ 3 mm, and that PGW was not an independent risk factor for PEP in patients with pancreatic duct diameter > 3 mm. To our knowledge, there has been no study focusing on the relationship between pancreatic duct diameter and PEP, and no prospective randomized study on this subject. We performed a new attempt of creating quasi-randomization using the IPTW method by propensity score to minimize bias from confounding variables [19]. This is the first report using IPTW to compare the relationship of PGW to PEP, focusing on pancreatic duct diameter.

PGW was performed in difficult cannulation cases, and the rate of PEP after PGW was reported to be 4.6–38% [4, 9, 20]. In this study, the rate of PEP was 21.1% (12/57) corroborated with previous reports. The mechanism of PEP is still unclear, although it is considered to occur due to various factors [21, 22]; (1) Increased inner pressure of the pancreatic duct caused by postoperative papilledema; (2) Mechanical irritation such as a damaged pancreatic duct due to a device, such as the guidewire; (3) Hydrostatic injury due to frequent pancreatography, manometry, or reflux water of pancreatoscopy; (4) Chemical injury due to infusion of the contrast medium or intestinal juice into the pancreatic duct; And (5) thermal injury due to papilledema by radiofrequency radiation or thermal damages of the pancreas itself. PGW may increase the risk of PEP because stimulation and/or pressure are added to the pancreatic duct. There were many reports investigating the relationship between PGW and PEP. According to reports from randomized controlled trials (RCTs), there was no significant difference between PGW and persistent attempts with conventional cannulation techniques regarding PEP [10, 11]. One RCT concluded that precut sphincterotomy increases the risk of PEP [9], whereas another concluded that it does not [4]. The latest meta-analysis of seven RCTs showed that PGW appeared to increase the risk of PEP relative to other techniques. However, in two reports, pancreatic duct stent was found to affect the risk of PEP [20, 23], and in four of the other five reports, there was no significant difference in the risk of PEP [24]. Following these results, the effect of PGW on the risk of PEP remains highly controversial. We think PGW may increase the risk of PEP by stimulating and/or pressuring the pancreatic duct. Actually, it was reported that even inadvertent cannulation of the pancreatic duct was associated with an increased risk of PEP [25, 26]. If there is a relationship between PEP, pancreatic duct pressure, and mechanical irritation as described above, pancreatic duct diameter would be an important predictive factor for PEP, because in patients with a small pancreatic duct diameter, the pancreatic duct pressure of a small duct may rise more easily due to narrowing or obstruction of the pancreatic orifice by the guidewire and papilledema from multiple manipulations during ERCP than that of a dilated pancreatic duct. Furthermore, a dilated pancreatic duct might be more tolerant to stimulation and pressure than a small pancreatic duct due to the constant load applied to the pancreatic duct by congestion of pancreatic juice. Concerning mechanical irritation to the pancreatic duct, there would be less guidewire friction and fewer guidewire-duct contacts during ERCP in a dilated duct than in a small duct. Based on our hypothesis, a narrow pancreatic duct diameter was considered to be a risk factor of PEP. Hence, this study used pancreatic duct diameter to define a new subject population as defined in other papers [13–15]; patients with a pancreatic duct diameter of ≤ 3 mm. Further, we analyzed another population who included 108 patients with a pancreatic duct diameter > 3 mm. The results of analysis of the two groups (Table 3) might back up our hypothesis that PGW increases the risk of PEP in patients with a pancreatic duct diameter of ≤ 3 mm, but not in patients with a pancreatic duct diameter > 3 mm.

The increased risk of PEP after PGW in patients with a pancreatic duct diameter of ≤ 3 mm necessitates new techniques to treat patients who have been recommended for PGW as a solution for difficult cannulation. According to some reports, a pancreatic duct stent placement has been effective in preventing PEP [21, 27, 28]; the benefit is attributed to the maintenance of a drainage route when papilla are blocked as a result of edema or spasm of the sphincter of Oddi, or both [29]. Ito *et al*. reported that PEP in patients who underwent PGW can be prevented by pancreatic duct stent placement [23, 30, 31]. It is suggested that each patient’s pancreatic duct diameter should be measured before ERCP; if the pancreatic duct diameter is ≤ 3 mm, PEP may be prevented by the insertion of a pancreatic duct stent at the end of ERCP.

There were several limitations to this study. First, while propensity score analysis, a statistical method of adjusting for selection bias in observational studies, approximates randomized trial approaches, this method has inherent limitations, namely, the choice of a finite number of covariates, which allows the possible omission of relevant covariates. Nonetheless, we believe that the most likely confounders were identified in our study, although we recognize that it is difficult to adjust for potential confounders using propensity score analysis. Second, we could not evaluate the relationship of the risk of PEP to procedure time, cannulation attempt number, number of times the guidewire was inserted into the pancreatic duct, number and amount of opacification of the pancreatic duct, history of sphincter of Oddi dysfunction, or guidewire size, because this study was retrospective and the information was unavailable. Regarding the use of NSAIDs, no patients used prophylaxis of NSAIDs suppository before or after ERCP, because prophylaxis of NSAIDs in Japan was not reimbursed by insurance company currently and we couldn’t collect data about history of using oral NSAIDs. Third, we measured pancreatic duct diameter using varying images: non-contrasted CT, enhanced CT and MRI. Fourth, we analyzed the risk of PEP in the population of patients with duct diameters > 3 mm as another-model in Table 3b, but the population was not large enough for statistical significance. Fifth, this report’s external validity was low because of its retrospective and single-center nature. Further research with a multicenter study may overcome this limitation.

In conclusion, PGW increases the risk of PEP in patients with pancreatic duct diameter of ≤ 3 mm; in these patients, further precautions should be taken during ERCP to prevent pancreatitis.

## Supporting information

S1 Data Data of 332 patients. S1 Data shows all the data of 332 patients.
(XLSX)

S2 Data Data of 108 patients. S2 Data shows all the data of 108 patients.
(XLSX)
